# Novel Temperature‐Sensitive Hydrogel Promotes Wound Healing Through YAP and MEK‐Mediated Mechanosensitivity

**DOI:** 10.1002/adhm.202201878

**Published:** 2022-10-06

**Authors:** Ze Li, Jinjian Huang, Yungang Jiang, Ye Liu, Guiwen Qu, Kang Chen, Yun Zhao, Peige Wang, Xiuwen Wu, Jianan Ren

**Affiliations:** ^1^ Lab for Trauma and Surgical Infections Department of Surgery Affiliated Jinling Hospital Medical School of Nanjing University 305 East Zhongshan Road Nanjing 210002 P. R. China; ^2^ Department of Emergency Medicine The Affiliated Hospital of Qingdao University 26 Jiangsu Road Qingdao 266000 P. R. China; ^3^ School of Medicine Southeast University Nanjing 211189 P. R. China; ^4^ Department of General Surgery, BenQ Medical Center The Affiliated BenQ Hospital of Nanjing Medical University Nanjing 210021 P. R. China; ^5^ Gastrointestinal Unit and Center for the Study of Inflammatory Bowel Disease Massachusetts General Hospital Harvard Medical School Boston MA 02114 USA

**Keywords:** contraction, hydrogels, MEK, temperature‐sensitive hydrogels, wound healing, YAP

## Abstract

Wound healing is a significant problem in clinical management. Various functional dressings are studied to promote wound healing through biochemical factors. They are generally expensive, complex to fabricate, and may adversely affect the wound. Mechanical forces are the critical regulators of tissue repair. Although contraction is shown to promote wound healing, the underlying mechanisms are not fully understood. In this study, a novel adhesive temperature‐sensitive mechanically active hydrogel with a simple and inexpensive preparation process is developed. The dressing is able to adhere to the wound surface and actively contract the wound in response to body temperature. This mechanical contraction enhances the proliferative activity of basal cells, reduces the inflammatory response of the wound, and promotes wound healing. Furthermore, RNA‐seq clarifies how the gene regulatory network is regulated by contraction. Finally, using pharmacological inhibitors, YAP and MEK are identified as the key signaling molecules for contraction‐mediated tissue healing in vivo.

## Graphical abstract

A novel viscous temperature‐sensitive mechanoactive hydrogel is designed, optimized, and characterized. Mechanical contraction is capable of promoting wound healing by enhancing the proliferative activity of basal cells. The typical mechanically stimulated transducers (YAP and MEK) are indispensable in the mechanical contraction‐mediated wound healing process.

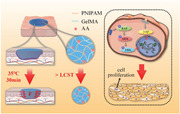

## 1 Introduction

Wound healing is a significant problem in clinical management. The annual medical expenditure on wound healing is well over ten billion dollars, resulting in a significant economic burden. The key to wound management is achieving efficient wound healing.^[^ ^1^, ^2^ ^]^ For wounds where the edges can be attached, sutures and staples are often used to promote healing. However, this can cause damage to the surrounding skin tissue, leading to complications such as scar formation, inflammation, and infection. Worse still, these methods are not applicable to severe tissue defects, and only dressings can be used to protect the wound.

Gauze is commonly used clinically as a dressing to keep wounds clean and dry, but its healing rate, healing quality, and adhesion to granulation tissue are not satisfactory.^[^ ^3^ ^]^ Research has been devoted to the study of various functional wound dressings that protect wounds, prevent infection, accelerate cell proliferation, and improve tissue remodeling.^[^ ^4^, ^5^, ^6^, ^7^ ^]^ The most frequently used strategies include the slow release of growth factors, loaded antimicrobial particles, loaded cells, modulation of local inflammatory response, removal of oxygen free radicals, the provision of adequate oxygen supply to the wound, and other biochemical approaches.^[^ ^8^, ^9^, ^10^, ^11^, ^12^, ^13^ ^]^ These dressings, however, are usually expensive and complicated to fabricate. In addition, the cells, cytokines, and functional components loaded in a wound dressing are usually effective only at specific time, and at other times they may even play the opposite role.^[^ ^14^ ^]^

Mechanical forces are the critical regulators of tissue repair.^[^ ^15^ ^]^ Cells in vivo transmit and withstand internal and external mechanical forces through cell–cell adhesion and interaction with the extracellular matrix (ECM).^[^ ^16^ ^]^ This, in turn, regulates the activation of biochemical signaling pathways and downstream gene transcription, ultimately affecting cell fate.^[^ ^17^, ^18^ ^]^ A synergistic strategy based on increased stiffness and production of reactive oxygen species (ROS) was found to promote the directed differentiation of bone‐marrow‐derived stem cells into cartilage and promote defective tissue repair.^[^ ^19^ ^]^ Mariaceleste reported that mechanical stretching promotes the expansion of intact skin by affecting the gene regulation of skin stem cells.^[^ ^20^ ^]^ In clinical practice, we also have found that the closure of the abdominal cavity in patients with open abdomen can be accelerated by constant tightening of the temporary abdominal closure (TAC) device. Accelerating wound healing by mechanical contraction is more effective, but mechanical contraction‐mediated tissue repair has been studied less often.

In this study, we developed a novel adhesive temperature‐sensitive mechanically active hydrogel dressing, based on methacrylic anhydride modified gelatin (GelMA), N‐isopropylacrylamide (NIPAM), and acrylic acid (AA). The preparation of the hydrogel was simple, efficient, and economical. The dressing was capable of adhering to the wound surface and contracting actively in response to body temperature, which mediated the contraction of the tissue surrounding the wound. In this study, we evaluated the therapeutic efficacy of active contraction hydrogel dressings in promoting wound healing. We also identified the underlying mechanisms by which active contraction of hydrogel dressings can promote wound healing. Previous studies have shown that actively contracting hydrogels are able to regulate the polarization of macrophages from a pro‐inflammatory phenotype (M1 type) to an anti‐inflammatory phenotype (M2 type) to promote wound healing.^[^ ^1^, ^21^, ^22^ ^]^ However, the molecular mechanism is not fully understood. We investigated the molecular characterization of mechanical contraction‐mediated wound healing to reveal key signaling molecules in the contraction‐mediated tissue healing process in vivo.

## 2 Results and Discussion

### 2.1 Preparation and Characterization of GNA Hydrogel

**Figure 1:**
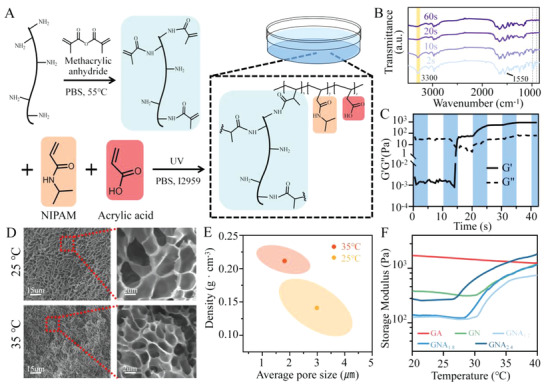
Synthesis of GNA hydrogel and thermoresponsive behavior. A) Schematic diagram of synthetic GNA hydrogel. B) Fourier transform infrared spectra of GNA hydrogels with different UV irradiation times. C) The photosensitivity and gel formation time of GNA hydrogel revealed by the rheological test. D) SEM images of GNA hydrogels at different temperatures. E) Density and pore size of GNA hydrogels at different temperatures. F) The effects of NIPAM and AA on hydrogels were validated by rheological tests, the GNA subscript numbers are the final concentration of acrylic acid (v/v, %).

We prepared the GNA hydrogel by mixing GelMA, NIPAM, and AA, using the Michael addition reaction (**Figure** **1**A). The network of PNIPAM has temperature‐sensitive properties capable of mechanically contracting the wound. Meanwhile, GelMA contains arginine‐glycine‐aspartate sequences (RGD) and matrix metalloproteinase target sequences (MMP) that promote cell adhesion and remodeling.^[^ ^23^ ^]^ In addition, polyacrylic acid (PAA) exhibits strong muco‐adhesive properties and good biocompatibility, and it is often used as a component of medical glue in clinical practice.^[^ ^24^, ^25^ ^]^ The combination of these properties enabled the GNA hydrogel to undergo appropriate active shrinkage at 35 °C (Movie S1, Supporting Information).

We obtained GelMA through a chemical modification of gelatin with methacrylamide (MA) side groups. The MA substituents were determined according to the method of Aleksandr.^[^ ^26^ ^]^ GelMA, with 66.91% substitution, was selected for follow‐up study (Figure S1A, Supporting Information). Fourier transform infrared spectroscopy (FTIR) spectra confirmed the synthesis of GNA hydrogels. The FTIR spectrum of GNA showed a significant enhancement of the stretching vibration of the carbonyl group after the addition of AA (Figure S1B, Supporting Information). The peak at 1550 cm^−1^ shown in Figure 1B was the characteristic peak of the amide bond. In addition, the peak at 3300 cm^−1^ was attributed to the stretching vibration of =C—H, and this peak disappeared after 20 s of ultraviolet (UV) irradiation. Similarly, the storage modulus of GNA hydrogels increased under UV irradiation (Figure 1C) and stabilized after 20 s. Therefore, we set the UV irradiation time to 20 s.

### 2.2 Thermoresponsive Behavior of GNA Hydrogel

**Figure 2:**
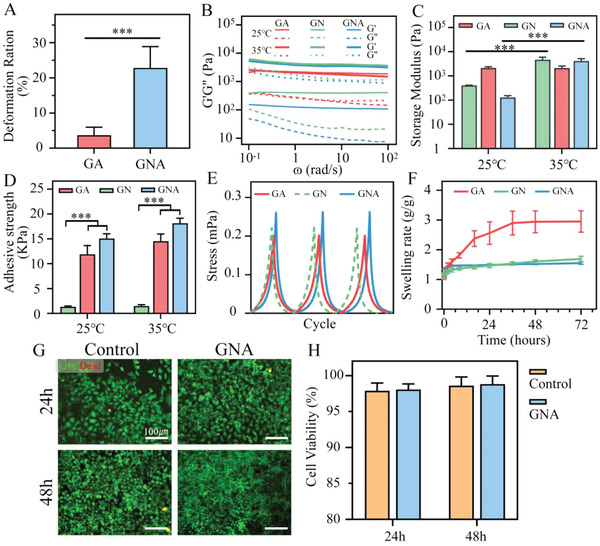
Material characteristics of GNA, GN, and GA hydrogels A) Deformation rate of GA and GNA hydrogels at 35 °C (*n* = 3). B,C) Frequency‐dependent oscillatory shear rheology of hydrogels at different temperatures (*n* = 3). D) Adhesive force test. AA improves the adhesion properties of hydrogels (*n* = 3). E) Compression testing. GNA, GN, and GA hydrogels have good fatigue resistance. F) Swelling ratios of GNA, GN, and GA hydrogels at 35 °C (*n* = 3). G,H) Live/Dead staining after co‐culture of fibroblasts with GNA hydrogel and its leachate (*n* = 3). ***p < 0.001.

The lower critical solution temperature (LCST) of PNIPAM is 32 °C, but the copolymerization of hydrophilic monomers into the polymer will affect its LCST.^[^ ^27^ ^]^ We measured GNA hydrogel deformation, differential scanning calorimetry (DSC) curves, and stiffness using polymerizing GNA gels with different monomer concentrations at different temperatures (Figure 1F; Figure S2A–E, Supporting Information). Considering that the body surface temperature is lower than the core temperature,^[^ ^28^ ^]^ we selected 30% (w/v) NIPAM, 5% (w/v) GelMA, and 6% (w/v) AA as the reaction concentrations of GNA hydrogels. Scanning electron microscopy (SEM) images showed that the pore size of GNA hydrogels was significantly lower at 35 °C, compared with 25 °C, indicating GNA hydrogels exhibited good temperature‐sensitive contraction properties (Figure 1D,E). In addition, when compared with GA hydrogels without the PNIPAM network structure, GNA hydrogels contracted significantly at 35 °C, pulling the rat skin closer to the center (**Figure** **2**A; Movie S1, Supporting Information).

### 2.3 Material Characteristics of GNA, GN, and GA Hydrogels

For comparison, GA hydrogel and GN hydrogel (without AA) were synthesized using similar steps. The rheological behavior of the three different hydrogels showed that the storage modulus of GNA hydrogel and GN hydrogel was significantly higher at 35 °C, compared with the 25 °C environment, while the storage modulus of GA hydrogel remained constant (Figure 2B,C). GNA hydrogel had suitable adhesive strength, enabling to close the wound and pull the surrounding tissue (Figure 2D). The adhesive properties of GNA hydrogels were mainly due to the electrostatic and H‐bonding interactions between the hydrogel and the tissue interface.

GA, GN, and GNA hydrogels exhibited excellent compressibility and were able to withstand compressive stresses of more than 0.2 mPa (Figure S3A, Supporting Information). In addition to good fatigue resistance, the three hydrogels maintained compressibility and elasticity after applying 80% of the maximum compressive strain for three cycles (Figure 2E). The swelling rates of GNA and GN hydrogels showed significantly greater stability in wet conditions, compared with GA hydrogels (Figure 2F). This enabled the GNA hydrogels to provide stable shrinkage. Good cytocompatibility is the basis of hydrogel dressing application.^[^ ^29^ ^]^ In total, l929 fibroblasts were co‐cultured with GNA hydrogel and its leachate, which was found to have no acute cytotoxicity to fibroblasts. As shown in Figure 2G,H, live cells (green fluorescence) were spindle‐shaped, and fewer dead cells (red fluorescence) were visible in both the GNA and control groups.

### 2.4 GNA Hydrogel Induces Rat Wound Repair

**Figure 3:**
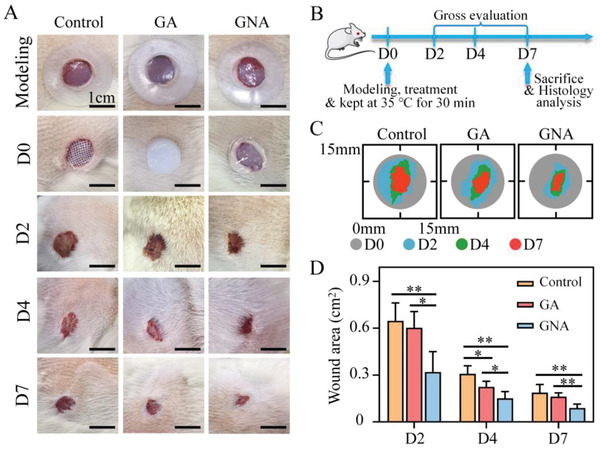
GNA hydrogel induces rat wound repair. A) Representative images of wound healing behavior in rats after different treatments. (The inner diameter of the circle is 1.2 cm). B) Experimental procedures. Groups of SD rats operated with total skin excision, given the corresponding treatment, and held at 35 °C for 30 min to promote active contraction of the hydrogel. Gross assessments were performed on day 2, 4, and 7. Rats were sacrificed on day 7 for pathological analysis (*n* = 5). C,D) Quantitative measurement of wound area in different treatment groups. *p < 0.05 and **p < 0.01.

In this study, we investigated the effect of GNA and GA hydrogels on tissue repair using a rat model with full‐thickness skin wounds. As shown in **Figure** **3**A,B, rats were placed into three treatment groups: 1) conventional gauze dressing (Control), 2) GA hydrogel, and 3) GNA hydrogel. The treated rats were kept at 35 °C for 30 min to promote active contraction of the GNA hydrogel. Wound healing was assessed on day 2, 4, and 7 after injury.

Macroscopic wound closure was sufficient in each group. Wound closure was significantly better in the GNA group than in the other two groups (Figure 3A,C,D). The supracellular actomyosin contractility of cells near the wound edge exerted sufficient tension on the surrounding tissue to promote wound closure.^[^ ^30^ ^]^ The GNA hydrogel was capable of promoting wound healing on a macroscopic level by actively contracting the tissue around the wound.

**Figure 4:**
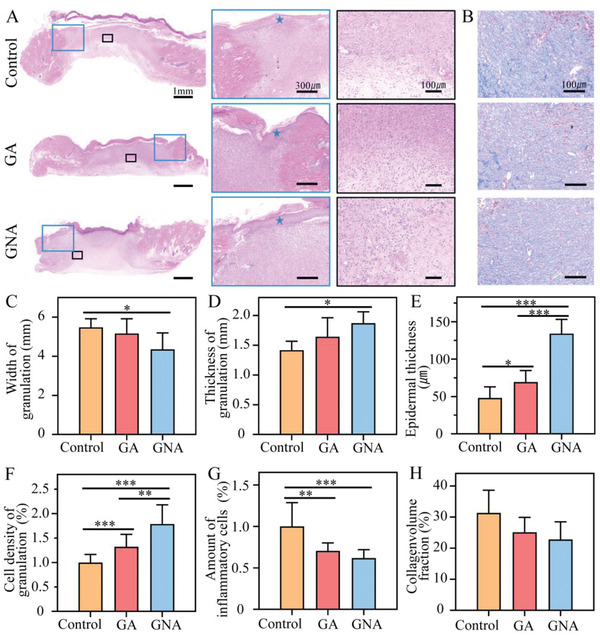
Histological evaluation of the wound at day 7. A) H&E staining, the blue pentagrams indicate the epidermis (*n* = 5). B) Masson's stain (*n* = 5). C–H) Quantitate analysis of wound healing quality. C) Width of the granulation. Three positions were taken for each tissue to measure the distance between the muscle layers on both sides and averaged. D) Thickness of the granulation. The distance from the basal lamina to the bottom was measured at 3 positions for each tissue and averaged. E) Epidermal thickness. The epidermal thickness of the wound closure site was calculated. F) Cell density of granulation. Based on the number of cells per unit area. G) Number of inflammatory cells. By analyzing the number of inflammatory cells per unit area. H) Collagenvolume fraction. The amount of collagen per unit area was analyzed by Masson's staining. *p < 0.05, **p < 0.01, and ***p < 0.001.

Histological evaluation of the neonatal wound tissue further supported the ability of the GNA hydrogel to significantly promote wound repair through active contraction. **Figure** **4**A shows representative images of the wound bed stained with hematoxylin and eosin (H&E), as well as localized magnified images. The width of the wound granulation tissue was smaller and the thickness was greater in the GNA group compared with the control group (Figure 4C,D). This change was attributed mainly to the active contraction of the GNA, which reduced the wound area. The high quality of early wound healing also was reflected in the thicker epidermis. Wounds in all three groups showed varying degrees of re‐epithelialization after 7 days, with the highest level of wound re‐epithelialization seen in the GNA group (Figure 4A,E). In addition, as shown in Figure 4F, the GNA group had a higher cell density.

In Figure 4A,G, the infiltration of inflammatory cells in the wounds indicated different degrees of inflammation in the wounds of the three groups, with the GNA hydrogel group having the least inflammatory cells in the wounds. In addition, we did not observe any significant difference in the collagen volume of the wounds in the three groups, but the GNA group was arranged in a more regularly fashion (Figure 4B,H). This arrangement indicated that the GNA hydrogel provided a suitable healing environment for wounds, and the healing of wounds in the GNA group resembled that of normal skin.

### 2.5 Contraction Promotes Basal Cell Proliferation

**Figure 5:**
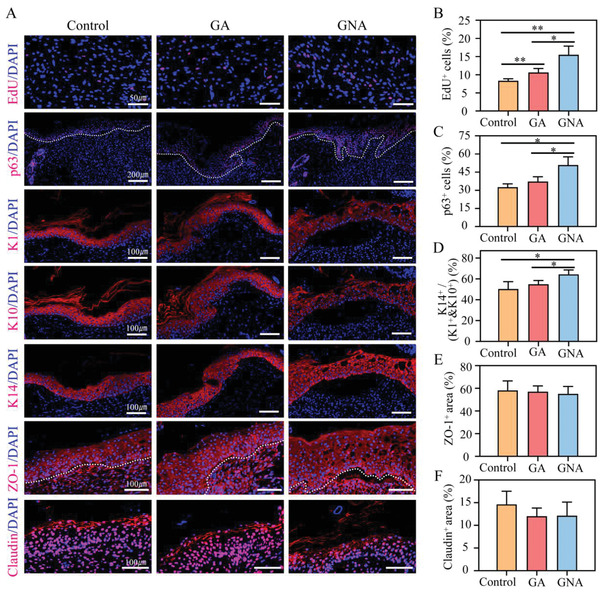
GNA hydrogel upregulates the proliferative activity of wound basal cells. Immunofluorescence staining for Edu, p63, keratin 1, keratin 10, keratin 14, zo‐1 and Claudin for the wound at day 7 (*n* = 5). B–F) Immunofluorescence signal quantification. B) Quantitative analysis of EDU^+^ cells. Percentage of EdU^+^ cells (red) among all DAPI^+^ cells (blue). C) Quantitative analysis of p63^+^ cells in the epidermis. D) Quantitative analysis of K14^+^/(k1^+^+k10^+^) cells. k14 is a marker of skin basal cell proliferation. k1 and k10 are markers of skin basal cell differentiation. The ratio of the two reflects the proportion of proliferating and differentiated cells in the wound epidermis. E,F) Quantification of fluorescence signal area of ZO‐1 and Claudin in the epidermis. *p < 0.05 and **p < 0.01.

Immunofluorescence data on wound neoplastic tissue demonstrated a onefold increase in 5‐ethynyl‐2‐deoxyuridine (EdU) incorporation after application of actively contracting GNA hydrogel (**Figure** **5**A,B). As previously reported, p63 is a master regulator of epidermal development and can mediate basal cell proliferation.^[^ ^31^ ^]^ The expression of p63 in basal cells was significantly increased in the GNA group (Figure 5A,C). Keratin is mainly expressed by epidermal cells, keratin 1 (K1) and keratin 10 (K10) are markers of early cell differentiation, and cells with proliferative activity express keratin 14 (K14).^[^ ^20^, ^32^ ^]^ The effect of active contraction of GNA hydrogels on the proliferation and differentiation of wound neoplastic cells was further assessed by analyzing the expression of K1, K10, and K14 in neoplastic epidermal cells. The K14^+^/(K10^+^&K10^+^) ratio was significantly increased on the basal layer of the GNA group. Moreover, the expression of tight‐junction proteins was unchanged (Figure 5A,E,F). These results demonstrated that contraction promoted the proliferation of basal cells around the wound.

### 2.6 Molecular Characteristics of Contraction

**Figure 6:**
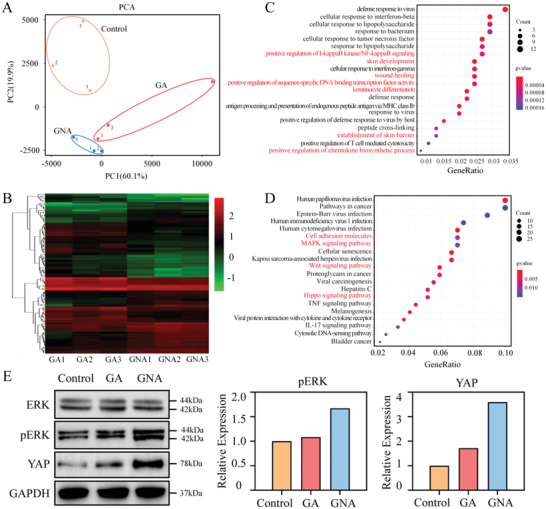
RNA sequencing of rat wound granulation tissue. A) Principal component analysis (PCA) for wounds in the control, GA and GNA groups after 7 days of treatment. B) Hierarchical clustering analysis of differentially expressed genes in wound granulation tissue. C) Gene ontology analysis. Differentially expressed genes were enriched in the GNA group compared to the GA group in regulating cell proliferation, differentiation and tissue repair. D) KEGG pathway enrichment analysis. The differentially expressed genes in the GNA group were enriched in cell adhesion, proliferation, differentiation, migration, and death related pathways compared to the GA group. E) Western blots and quantitative analysis of wounds in the control, GA and GNA groups after 7 days of treatment.

To define the gene expression changes in contraction‐mediated wound healing, we performed RNA sequencing and analysis of neonatal tissue from rat wounds. Principal component analysis (PCA) of gene expression profiles revealed significant differences in gene expression among the three groups of neonatal tissues (**Figure** **6**A). Further stratified analysis of differentially expressed genes (DEGs) showed that 187 genes were upregulated and 374 genes were downregulated in the GNA group, compared with the GA group (Figure 6B). This result demonstrated that actively contracting hydrogels could affect cellular transcription during wound healing.

Gene ontology (GO) enrichment analysis showed that genes promoting cell proliferation, such as positive regulation of I‐kappaB kinase/NF‐kappaB signaling, wound healing, positive regulation of sequence‐specific DNA‐binding transcription factor activity, and positive regulation of chemokine biosynthetic process, were upregulated in the GNA group, and genes regulating cell differentiation, such as keratinocyte differentiation, skin development, and establishment of skin barrier, were downregulated (Figure 6C; Figure S3B, Supporting Information). Moreover, Kyoto Encyclopedia of Genes and Genomes (KEGG) pathway enrichment analysis revealed that cell adhesion molecules, the mitogen‐activated protein kinase (MAPK) signaling pathway, the Wnt signaling pathway, and the Hippo signaling pathway were involved in the contraction‐mediated wound repair process (Figure 6D; Figure S3C, Supporting Information). It has been reported that cell adhesion molecules are the most important regulators of the Hippo pathway, and the Wnt pathway interacts with the Hippo pathway.^[^ ^33^ ^]^ This interaction suggests that active contraction of GNA hydrogels could promote wound healing regulation through the MAPK and Hippo pathways. These results were also confirmed by western blot (Figure 6E).

### 2.7 MEK/ERK and YAP Regulate Skin Healing

Previous studies have shown that mechanical stimulation affects cell fate by activation of the yes‐associated protein (YAP), a downstream co‐effector of Hippo signaling, and mitogen‐activated protein kinase (MEK), which is part of the MAPK pathway.^[^ ^34^, ^35^ ^]^ The mechanical strain induced YAP translocation from the cytoplasm to the nuclei, which, in turn, increased the proliferation and differentiation of isolated cells.^[^ ^36^ ^]^ ERK can be activated through changes in cell membrane tension, and it is involved in the regulation of diverse cellular functions, including adhesion, migration, cell cycle progression, cytokinesis, proliferation, and differentiation.^[^ ^37^, ^38^ ^]^

**Figure 7:**
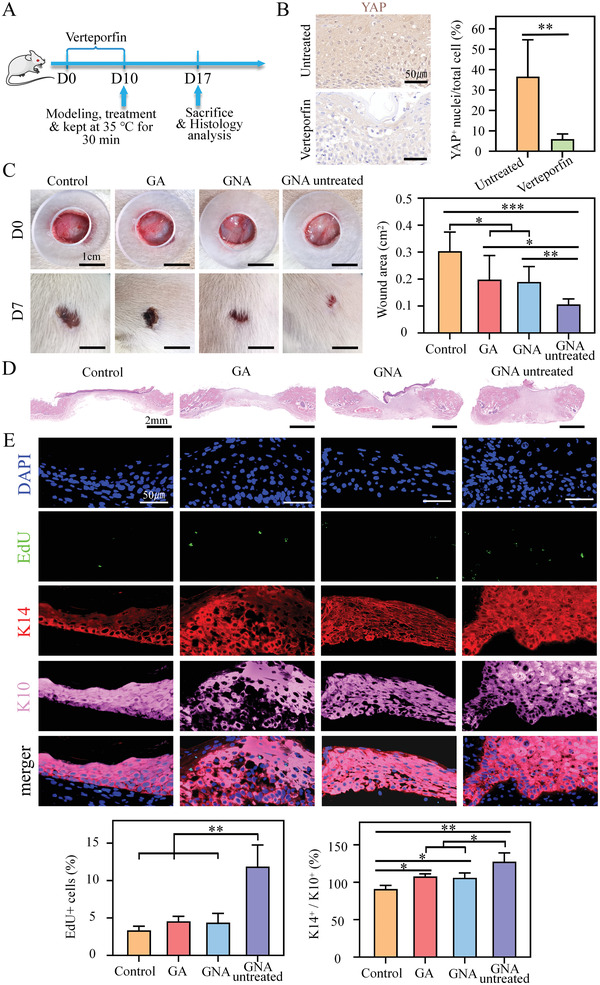
YAP regulates contraction‐mediated proliferation. A) Experimental procedure. Protocol for Verteporfin treatment in SD rats operated in full skin excision after treatment and evaluated. B) YAP immunostaining and quantification on skin sections of control and Verteporfin treated groups (*n* = 5). C) Representative images and quantification of YAP‐inhibited rat wound healing behavior by gauze, GA hydrogel and GNA hydrogel. D) Representative images of HE staining of wound granulation tissue. E) Representative images and quantification of epidermal immunofluorescence staining of wound closure sites (*n* = 5). *p < 0.05 and **p < 0.01.

**Figure 8:**
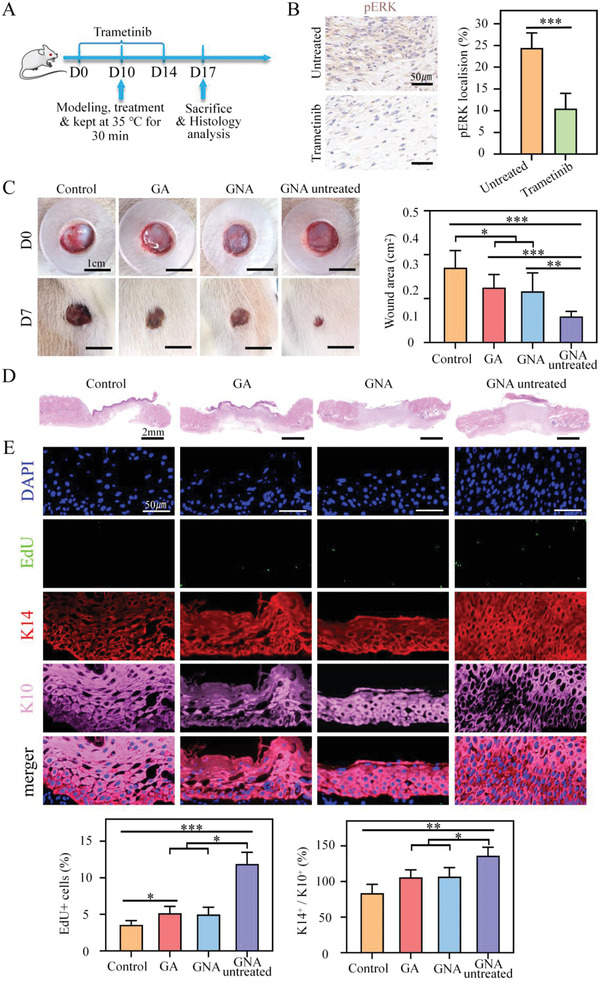
A) MEK/ERK regulates proliferation. Experimental procedure. Protocol for Trametinib treatment in SD rats operated in full skin excision after treatment and evaluated. B) pERK immunostaining and quantification on skin sections of control and Trametinib treated groups (*n* = 5). C) Representative images and quantification of MEK‐inhibited rat wound healing behavior by gauze, GA hydrogel and GNA hydrogel. D) Representative images of HE staining of wound granulation tissue. E) Representative images and quantification of epidermal immunofluorescence staining of wound closure sites (*n* = 5). *p < 0.05, **p < 0.01, and ***p < 0.001.

To assess whether pharmacological inhibition of the YAP and MEK/ERK pathways could impair cellular behavior after skin contraction, we treated rats with the YAP inhibitor, verteporfin, and the MEK inhibitor, Trametinib (**Figures** **7**A and **8**A). The inhibitor downregulated YAP expression and reduced its nuclear translocation (Figure 7B). Trametinib reduced pERK (Figure 8B). In both inhibition models, we found that the effect of actively contracting GNA hydrogels to promote wound healing was significantly diminished, and there were no differences in wound closure, granulation tissue thickness, EdU incorporation, and K14+/k10+ ratio between the GNA hydrogel and GA hydrogel groups (Figures 7C–E and 8C–E). Inhibition of YAP or MEK blocked the contractile response of the wound, suggesting that YAP and MEK/ERK signaling regulate epidermal cell renewal in contraction‐mediated wound healing in vivo.

## 3 Conclusion

In conclusion, we developed a novel adhesive mechanically active hydrogel dressing to accelerate wound healing. Compared with the control, the GNA hydrogel treatment mechanically contracted the wound, reduced inflammation, and improved wound healing quality. During the contraction‐mediated acceleration of wound healing, there was a transient shift of basal cell fate toward renewal. These results were supported by RNA‐seq, which revealed the molecular signature of mechanical contraction‐mediated tissue healing and identified the potential molecular mechanisms of mechanical contraction‐mediated higher proliferative activity in rat dorsal wounds. Furthermore, we showed that the typical mechanically stimulated transducers (YAP and MEK) were indispensable in the contraction‐mediated wound healing process. Interestingly, these signals were activated during skin expansion in mice^[^ ^20^ ^]^ and during embryonic pancreas development,^[^ ^39^ ^]^ suggesting a conserved role in tissue regeneration and embryonic development.

## 4 Experimental Section

### Materials, Cell Lines, and Animals

AA, 2‐hydroxy‐4'‐(2‐hydroxyethoxy)‐2‐methylpropiophenone (I2959), MA, gelatin, and NIPAM were purchased from Sigma–Aldrich (St. Louis, MO, USA). 5‐Ethynyl‐2‐deoxyuridine (EdU) was purchased from Wuhan Servicebio Technology Co., Ltd. (Hu Bei, China). Trametinib was purchased from ChemeGen Biotechnology Co., Ltd. (Shanghai, China). Verteporfin was purchased from Aladdin Co., Ltd. (Shanghai, China). All other reagents were of analytical reagent grade.

L929 fibroblast cells (KeyGEN BioTech Co., Ltd., Jiangsu, China) were cultured in Dulbecco's modified eagle medium (DMEM; Servicebio) containing 10% fetal bovine serum (FBS; OriCell, Shanghai, China) and 1% penicillin/streptomycin (Servicebio) at 37 °C in a moist atmosphere (5% CO_2_, 95% air).

Male Sprague Dawley rats (7 weeks) were provided by Jinling Hospital and were anesthetized using intraperitoneal injections of 20% urethane (Macklin, Shanghai, China) at 1 g kg^−1^. The rats were kept under a natural light–dark cycle at 25 °C, with free access to food and water. All animal experiments were performed in compliance with the Chinese guideline for the care and use of laboratory animals (Ministry of Science and Technology [2006] file no. 398) and approved by the Institutional Animal Care and Use Committee of Jinling Hospital (approval number 2022DZGKJDWLS‐0074).

### Synthesis of GelMA

GelMA was synthesized as previously described.^[^ ^40^ ^]^ Briefly, 150 mL of gelatin aqueous solution (8%, w/v) was prepared by dissolving gelatin powders into Dulbecco's phosphate‐buffered saline (DPBS) at 60 °C and stirring for 3 h. Three milliliters of MA was added at a rate of 0.3 mL min^−1^, allowing the reaction to proceed for 3 h at 55 °C. To stop the methacrylation reaction, 150 mL of warm DBPS was added, and the reaction was dialyzed for 7 days using dialysis membranes (molecular weight cutoff: 12–14 kDa). Deionized water was replaced every 8 h. Finally, the dialyzed solution was lyophilized.

### Preparation of GNA, GN, and GA Hydrogels

GelMA, NIPAM, and AA were dissolved in PBS containing 0.5% (w/v) I‐2959 to obtain solutions with different concentrations, and the solutions were mixed in equal proportions. The mixed solutions were bubbled under N_2_ for 30 min to remove oxygen. The resulting solutions were photoinitiated under UV light exposure (365 nm, ≈6.0 W cm^−2^; Automatic, FUTANSI Electronic Technology Co., Ltd., Shanghai, China) at 4 °C. After gelation, the hydrogels were soaked in PBS for 30 min and rinsed three times before use. The GN, GA hydrogels were fabricated through a similar procedure.

### Characterization

The grafting rate of MA was confirmed, and the nuclear magnetic resonance (NMR) spectra of GelMA dissolved in deuterated solvents were characterized by ^1^H NMR (Bruker, Karlsruhe, Germany). Subsequently, the gelling time and thermosensitive behavior of GNA hydrogels were determined. FTIR spectra of GelMA, NIPAM, and GNA hydrogels with different UV exposure times were studied at room temperature using a Nicolet‐6700 spectrometer (ThermoFisher Scientific, Waltham, MA, USA). Rheological parameters were measured by a rheometer (MCR302; Anton Paar GmbH, Graz, Austria), and the gap between the parallel plates was 1 mm. In the oscillatory time sweep experiment, the constant strain was fixed at 1%, and frequency was fixed at 10 Hz. Five on‐and‐off cycles of UV light were applied on the GNA solutions, with a cycle duration of 10 s, and the temperature was set to 25 °C. In addition, the oscillatory time sweep experiment of the GNA hydrogel was measured by increasing the temperature from 20 to 40 °C at a rate of 2 °C min^−1^. LCST was determined by subjecting the hydrogels to different ambient temperatures for 20 min, as determined by color and volume changes.

We observed the morphological structures of GNA hydrogels at 25 and 35 °C using an SEM (Phenom, Eindhoven, Netherlands). The density of the hydrogel was obtained by the ratio of the mass after lyophilization to the volume of the hydrogel at different temperatures. The volume was measured using the drainage method. As shown in Movie S1 (Supporting Information), the hydrogel was adhered to two pieces of rat skin at 35 °C for 30 min, and the change in length over time was measured. The initial and final lengths were recorded as L_O_ and L, and the deformation rate was calculated as 1 – L/L_O_. In the oscillatory frequency sweep experiment, the constant strain was fixed at 1%. For the adhesive force test, the adhesion strength of GNA, GN, and GA hydrogels to fresh porcine skin was investigated by a universal testing machine (MTSCMT2103; MTS Systems, Eden Prairie, MN, USA), as previously described.^[^ ^12^ ^]^ For compression testing, the hydrogel samples were prepared as cylindrical shapes with a diameter of 10 mm and a length of 8 mm. A preload force of 0.1 N was set on a testing machine (MTS Systems). The compression velocity was 5 mm min^−1^, and the stress value in the cyclic test was 80% of the maximum stress.

### Swelling Ratio

To determine the swelling ratio, hydrogels with a volume of 1 cm^3^ were immersed in 5 mL of PBS at 35 °C. The hydrogels were taken out at specific time points, and the surface water was wiped with a paper. They were weighed (M_T_) and recorded as soon as possible and then returned to the original PBS. The initial mass was M_I_, and the swelling ratio was (M_T_‐M_I_)/M_I_.

### Cytocompatibility Study

The biocompatibility of the cells was tested using a Live/Dead Cell Staining Kit (KeyGEN BioTech). L929 fibroblasts at a concentration of 0.5 × 10^5^ cells per well were seeded into 12‐well plates. After 24 h of incubation, 1 mL of hydrogel leachate was added to the experimental group, and 1 mL of DMEM medium was added to the control group. Incubation continued for 24 and 48 h. After the cells were stained, images were recorded by fluorescence microscopy.

### Wound Healing In Vivo

To verify the effectiveness of GNA hydrogel for wound healing, with reference to previous reports, the gel was adhered to a full‐thickness rat skin incision model. First, the dorsal area of the rat was shaved and disinfected with iodophenol and 75% alcohol. Then, a full‐thickness incision of 1.2 cm in diameter was created on both sides of the spine. All rats were randomly divided into three groups, and three different dressings were applied to the wound, including gauze, GA hydrogel, and GNA hydrogel. They were subsequently kept at 35 °C for 30 min. Finally, the wound closure was recorded at a preset time.

To inhibit YAP activity, rats were treated with 10 mg kg^−1^ verteporfin through intraperitoneal (IP) injection every other day before surgery for 10 days. For inhibition of MEK1/2 activity, rats received 0.3 mg kg^−1^ trametinib daily by oral gavage from 10 days prior to surgery to 4 days after surgery. As shown in the Figures 6A and 7A.

### Wound Tissue Microenvironment Assessment

Rats were executed 7 days after surgery. Their wound tissue was collected and fixed in 4% paraformaldehyde. H&E staining and Masson staining were performed immediately, and the width and thickness of the granulation tissue, the thickness of the epidermis, the density of cells, the number of inflammatory cells per unit area, and the fraction of collagen deposition were further analyzed by microscopy. EdU 5 mg kg^−1^ was injected intraperitoneally 24 h before sampling. Immunofluorescence staining for EdU, p63, K1, K10, K14, ZO‐1, and Claudin was performed after fixation of the wound tissue to assess the proliferation and differentiation of the granulation tissue, as well as the barrier function. The inhibitory effect was also assessed by immunohistochemistry for animal models injected with pharmacological inhibitors. Quantification of the stained tissue was performed using ImageJ. Each group was quantified using at least five images.

### RNA‐seq

Total mRNA samples were extracted from wound granulation tissue of rats at 7 days post‐operation by Trizol (Sigma–Aldrich), with three samples per group. These libraries were constructed using the VAHTS Universal V6 RNA‐seq Library Prep Kit for Illumina (NR604‐02; San Diego, CA, USA), and the constructed cDNA libraries were sequenced on the Illumina NovaSeq 6000 platform. Clean reads filtered from the raw reads were mapped to the reference using Hisat. Differential expression analysis was performed with the DESeqR software package (1.10.1). DESeq defined differentially expressed genes as those with FDR significance score <0.05 and absolute value of log_2_(FC) >1.5. GO and KEGG databases were enriched by the clusterProfiler R software package for DEG analysis. The raw data files of all RNA‐seq analyses were deposited in the SRA database (accession RJNA847699).

### Western Blotting

The proteins were separated by SDS‐PAGE and transferred to PVDF membranes. The membranes were then incubated with antibodies against pERK (abcam, ab201015), ERK (abcam, ab184699), YAP (abcam, ab76252), and beta Actin (abcam, ab8227) overnight at 4 °C. Positive signals were scanned using a Tanon‐4600 chemiluminescence imaging system (Tanon Science and Technology, China). The optical density of the protein bands was quantified with ImageJ and normalized by the expression of GAPDH.

### Statistical Analysis

Data were analyzed using GraphPad Prism (9.0). All data were shown as means ± SEM. Unpaired student's *t* test (two tailed) or one‐way analysis of variance (ANOVA) was used for comparison of two groups. Dunnett's or Bonferroni's post hoc test was used as needed. The thresholds for statistical significance were *p < 0.05, **p < 0.01, and ***p < 0.001.

## Conflict of Interest

The authors declare no conflict of interest.

## Supporting information

Supporting Information

Supplemental Movie 1

## Data Availability

The data that support the findings of this study are available from the corresponding author upon reasonable request.
