# Supplementary material for: Novel Temperature‐Sensitive Hydrogel Promotes Wound Healing Through YAP and MEK‐Mediated Mechanosensitivity
Source: Adv Healthc Mater. 2022 Oct 6;11(23):2201878. doi: 10.1002/adhm.202201878 (PMC11469276; doi:10.1002/adhm.202201878)
Supplement: Supplementary file 1 — Supporting Information [file ADHM-11-2201878-s002.pdf]

ADVANCED HEALTHCARE MATERIALS

Supporting Information

for *Adv. Healthcare Mater.*, DOI 10.1002/adhm.202201878

Novel Temperature-Sensitive Hydrogel Promotes Wound Healing Through YAP and MEK-Mediated Mechanosensitivity

Ze Li, Jinjian Huang, Yungang Jiang, Ye Liu, Guiwen Qu, Kang Chen, Yun Zhao, Peige Wang, Xiuwen Wu* and Jianan Ren**

Supporting Information

Novel temperature-sensitive hydrogel promotes wound healing through YAP and MEK-mediated mechanosensitivity

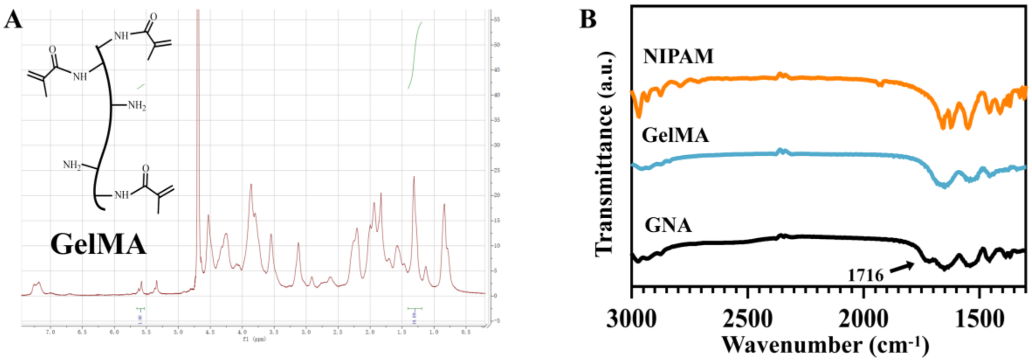

$$DM(\%)=0.3836mol/0.0385mol \times I_{5.7ppm}/I_{1.2ppm} \times 100=66.91$$

Figure S1. Characteristics of GelMA,GNA.

(A) NMR spectra of GelMA and formula for calculation of substitution degree. (B) Fourier transform infrared spectra of GNA hydrogels, GelMA hydrogels after lyophilization and nipam.

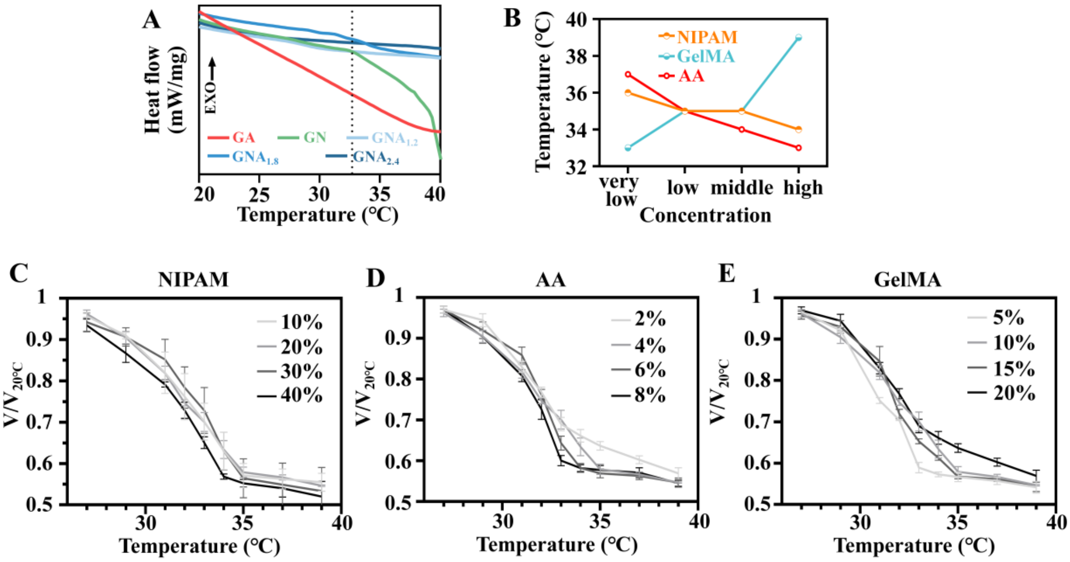

Table 1 Concentration

	very low	low	middle	high
**Nipam**	10	20	30	40
**AA**	2	4	6	8
**GelMA**	5	10	15	20

Figure S2. The lower critical solution temperature (LCST) of GA, GN, and GNA hydrogels.

(A) DSC curves of GA, GN and GNA hydrogels with different concentrations of AA after lyophilization. (B) LCST for different concentrations of GNA hydrogels (determined by combining color and area changes, concentrations as in Table 1. (C-E) Effect of different temperatures on the area of GNA hydrogels with different concentrations of NIPAM, AA and GelMA.

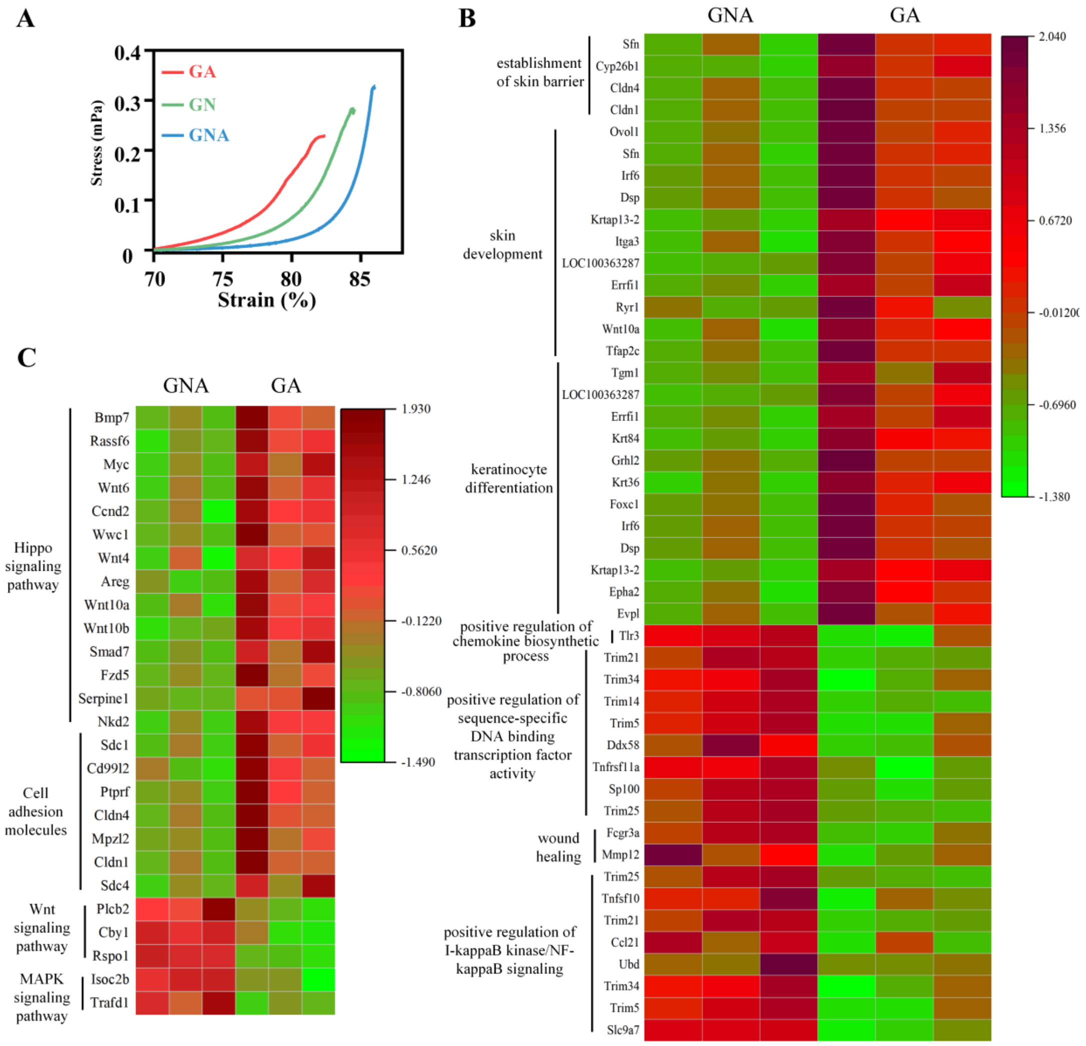

Figure S3. GA, GN and GNA hydrogel compression tests and RNA sequencing for differentially expressed gene enrichment analysis.

(A) GA, GN and GNA hydrogel compression tests. (B) Analysis of differentially expressed genes in the GA and GNA groups of SD rats after 7 d of wound treatment. The heat map shows that genes related to cell proliferation, migration and tissue healing were enriched in the GNA group, while genes related to differentiation and cell death were enriched in the GA group.
